# Improved Treatment of the Independent Variables for the Deployment of Model Selection Criteria in the Analysis of Complex Systems

**DOI:** 10.3390/e23091202

**Published:** 2021-09-11

**Authors:** Luca Spolladore, Michela Gelfusa, Riccardo Rossi, Andrea Murari

**Affiliations:** 1 Department of Industrial Engineering, University of Rome “Tor Vergata”, Via Del Politecnico 1, 00133 Roma, Italy; michela.gelfusa@uniroma2.it (M.G.); r.rossi@ing.uniroma2.it (R.R.); 2 Consorzio RFX (CNR, ENEA, INFN, Università di Padova, Acciaierie Venete SpA), Corso Stati Uniti 4, 35127 Padova, Italy; andrea.murari@istp.cnr.it

**Keywords:** model selection criteria, Akaike information criterion, Bayesian information criterion, overfitting, redundancy, variable selection, complexity, information theory, relevance

## Abstract

Model selection criteria are widely used to identify the model that best represents the data among a set of potential candidates. Amidst the different model selection criteria, the Bayesian information criterion (BIC) and the Akaike information criterion (AIC) are the most popular and better understood. In the derivation of these indicators, it was assumed that the model’s dependent variables have already been properly identified and that the entries are not affected by significant uncertainties. These are issues that can become quite serious when investigating complex systems, especially when variables are highly correlated and the measurement uncertainties associated with them are not negligible. More sophisticated versions of this criteria, capable of better detecting spurious relations between variables when non-negligible noise is present, are proposed in this paper. Their derivation is obtained starting from a Bayesian statistics framework and adding an a priori Chi-squared probability distribution function of the model, dependent on a specifically defined information theoretic quantity that takes into account the redundancy between the dependent variables. The performances of the proposed versions of these criteria are assessed through a series of systematic simulations, using synthetic data for various classes of functions and noise levels. The results show that the upgraded formulation of the criteria clearly outperforms the traditional ones in most of the cases reported.

## 1. Introduction to Model Selection Criteria Based on Bayesian Statistics and Information Theory

Model Selection (MS) can be defined as the task of identifying the model best supported by the data, among a set of potential candidates [1]. In many fields, model selection is an essential part of scientific enquiry [2]. It can also be argued that this step is often among the most delicate in statistical inference.

The exact definition of what is meant by the best model is controversial and is probably application dependent [3]. Indeed, the requirements of models are not the same if the goal of the study is prediction, explanation, or control. In any case, basically all approaches to model selection try to find a compromise between goodness of fit and complexity. At the same level of goodness of fit, simpler models, implementing some form of Occam’s razor, are preferred. The goodness of fit is assessed with the likelihood or, when this is not possible, with some metric quantifying the residuals, the distance between the model predictions, and the data. The complexity of the models is identified with the number of the model parameters. In the following, attention will be focussed on MSC derived with the help of Bayesian statistics and information theory, since these are the ones explicitly designed to find a trade-off between goodness of fit and complexity. In any case, similar considerations apply also to frequentist types of techniques. A remark about the nomenclature is in place at this point. Since the application covered in the present work is regression, with the term database in the following, it is indicated as a finite ordered list of entries. Each entry consists of a sequence of elements formed by a dependent variable, *y*, and a series of p regressors or predictors, *x_i_*.

The most widely accepted and best understood model selection criteria, based on information theory and Bayesian statistics, are the Akaike Information Criterion (AIC) [4] and the Bayesian Information Criterion (BIC) [5].

The theoretical derivations of these metrics result in the following unbiased forms of the criteria:

(1) $$AIC=-2\ln\left(L\right)+2k$$

(2) $$BIC=-2\ln\left(L\right)+k\ln\left(n\right)$$

where *L* is the likelihood of the model given the data, *k* the number of parameters in the model, and *n* the number of entries in the database (also called the sample size). Both *AIC* and *BIC* metrics are basically cost functions, which have to be minimized; they favour models with a high likelihood but implement a penalty for complexity (the term proportional to k).

Since in most applications, such as the ones discussed in this work, it is impossible to calculate the likelihood of the models, the metric adopted for the goodness of fit is the Euclidean distance of the residuals. Under the traditional assumption, that the data are identically distributed and independently sampled from a normal distribution, it can be demonstrated that the *AIC* can be written (up to an additive constant, which depends only on the number of entries in the database and not on the model) as follows:

(3) $$AIC=n\cdot \ln\left(MSE\right)+2k$$

where *MSE* is the mean-squared error of the residuals, *n* the number of entries in the database, and *k* the number of parameters in the model. Similar assumptions allow expressing the *BIC* criterion as follows:

(4) $$BIC=n\cdot \ln\left(\sigma_{\left(\epsilon \right)}{}^{2}\right)+k\cdot \ln\left(n\right)$$

where $\sigma_{\left(\epsilon \right)}{}^{2}$ is the variance of the residuals, *n* is again the number of entries in the database, and *k* the number of parameters in the model. The derivation of these two criteria in the various approximation is fully covered in [6].

These two indicators, and all the others belonging to the same families, are cost functions to be minimised, in the sense that the better the model the lower their value. This can be intuitively appreciated by a simple inspection of their structure. The first term favours models that are closer to the data. The second addend is the penalty term for complexity.

In the last years, various upgrades of these criteria have been proposed. They are mainly meant at improving the goodness of fit, by utilising more sophisticated statistics than the simple MSE, and at devising more accurate estimates of the penalisation for complexity [7,8]. All these improvements have proved to be quite significant, but they do not consider explicitly the problems related to the choice of the regressors and the effects of the measurement uncertainties. They basically assume that the independent variables have already been properly identified without any specific provision for this aspect. Some of them deploy quite sophisticated statistical indicators of the distribution of the residuals, but they all take the measurements as given without any error bar. These are all issues which can be quite relevant when investigating complex systems. Typically, in the field of complexity, various quantities can be spuriously correlated with the dependent one, and measurements can be affected by significant uncertainties due to the poor accessibility of many systems. In this situation, as will be shown in the following, the performance of the traditional versions of the AIC and BIC are unsatisfactory, both being prone to include redundant variables in the selected models.

This work aims to provide an upgraded version of the traditional AIC and BIC criteria to alleviate the problems posed by quantities spuriously correlated with the actual predictors. These quantities tend to mislead the available versions of the indicators, inducing them to converge on models with an excessive number of non-relevant regressors. The situation is significantly worsened by the presence of significant levels of noise, which tend to blur the relations between the dependent quantities and the predictors, as shown in Section 4, which is devoted to the numerical tests. It should be mentioned that the vast majority, if not all, of the applications of model selection criteria involve experimental measurements, which are always affected by some form of noise. The capability of the proposed improvements of dealing with uncertainties is therefore an important aspect that needs to be assessed.

The paper is organized as follows. In the next section, the main information theoretic indicators used in the rest of the paper are reviewed. In Section 3, the derivation of the upgraded version of the AIC and BIC is covered. In Section 4, the performances of the upgraded criteria are evaluated through a series of systematic tests. In Section 5, an application of the derived criteria to a real-life database is reported. The conclusions of the paper are presented in the final section.

## 2. Brief Review of the Information Theoretic Indicators Relevant to the Upgrades of the Model Selection Criteria

The first information theoretic quantity [9], required to understand the improvements of the MSC proposed in this work, is the Mutual Information (MI) between two random variables, *X* and *Y* [9]:

(5) $$MI\;\left(X_{i},Y\right)=-\sum_{X}\sum_{Y}P_{XY}\ln\left(\frac{P_{XY}}{P_{X}P_{Y}}\right)$$

where *P_XY_* is the joint probability distribution function (pdf) of the random variables *X* and *Y*. Being fully nonlinear, contrary to the Pearson correlation coefficient, the MI is well suited to extract, from a given database, the best features, i.e., the best regressors, *X_i_*, to reproduce the desired dependent variable *Y*.

The second important information theoretic indicator, used in the rest of the paper, is the concept of redundancy, RD, between a variable *X_i_* and a set, *S*, of other variables, *X_j_*:

(6) $$RD\left(X_{i},S\right)=\underset{X_{j}\in S}{\sum }MI\left(X_{i},X_{j}\right)$$

Mutual information and redundancy allow defining a quantity, called relevance RL, which quantifies the net contribution of a variable to reducing the uncertainty in a different one, *Y*, above what is already contributed by another set of quantities. Relevance is defined as

(7) $$RL\left(X_{i},Y\right)=MI\left(X_{i},Y\right)-RD\left(X_{i},S_{PS}\right)=MI\left(X_{i},Y\right)-\underset{X_{j}\in S_{PS}}{\sum }MI\left(X_{i},X_{j}\right)$$

## 3. Derivation of the Upgraded Version of the BIC and AIC

In this section, the original versions of the BIC and AIC criteria are reviewed, and this provides an introduction to the derivation of the upgraded versions of the criteria. The BIC criterion is discussed first because it allows a more natural introduction of the proposed improvements.

### 3.1. Upgraded Version of the BIC

The Bayesian approach to model selection is based on the maximization of the posterior probability of a model $M_{i}$ given the data $Y=y_{1},\ldots ,y_{n}$. From the Bayes theorem, this posterior probability can be written as follows:

(8) $$p\left(M_{i}|Y\right)=\frac{p\left(Y|M_{i}\right)\cdot p\left(M_{i}\right)}{p\left(Y\right)}\;$$

where $p\left(Y|M_{i}\right)$ is the marginal likelihood of the Model $M_{i}$ and can be evaluated as follows:

(9) $$p\left(Y|M_{i}\right)=\int L\left(Y|M_{i},\theta_{i}\right)\cdot f\left(\theta_{i}|M_{i}\right)d\theta_{i}$$

where $\theta_{i}$ is the vector of the parameters of the model $M_{i}\;$ and $f\left(\theta_{i}|M_{i}\right)$ is the probability distribution of the parameters.

It can be demonstrated that for high n, and setting $f\left(\theta_{i}|M_{i}\right)=1$ (uninformative prior), Equation (9) can be approximated with

(10) $$p\left(Y|M_{i}\right)\approx L\left(Y|M_{i},\hat{\theta_{i}}\right)\cdot e^{-\frac{\left|\hat{\theta_{i}}\right|}{2}\log n}\;\;\;\;\;$$

With $\hat{\theta_{i}}=argmax_{\theta_{i}\in \Theta }\left(L\left(Y|M_{i},\theta_{i}\right)\right)$.

Substituting (10) in (8), we obtain the following:

(11) $$p\left(M_{i}|Y\right)\propto L\left(Y|M_{i},\hat{\theta_{i}}\right)\cdot e^{-\frac{\left|\hat{\theta_{i}}\right|}{2}\log n}\cdot p\left(M_{i}\right)\;\;\;\;\;$$

If we set $p\left(M_{i}\right)=1$, which implies considering all the models equally probable, (11) leads to the traditional definition of the BIC. Indeed, after taking the logarithm and simple mathematical manipulations, (11) becomes the following:

(12) $$2\cdot \log\left(p\left(M_{i}|Y\right)\right)\approx 2\cdot \log\left(L\left(Y|M_{i},\hat{\theta_{i}}\right)\right)-\left|\hat{\theta_{i}}\right|\cdot \log n\;$$

The right-hand side of Equation (12) can be recognized as the BIC criterion estimate for the model $M_{i}$ with an inverted sign. Indeed maximizing (12) is equivalent to minimizing:

(13) $$BIC\approx -2\cdot \log\left(L\left(Y|M_{i},\hat{\theta_{i}}\right)\right)+\left|\hat{\theta_{i}}\right|\cdot \log n\;$$

In situations for which the relevant assumptions are valid, the likelihood can be replaced with the standard deviation of the residuals, with $\left|\;\hat{\theta_{i}}\right|$ as the number *k* of parameters in the model, allowing to recover Equation (4).

As shown in the following sections, when the redundancy between the regressors is not negligible, the traditional BIC criterion can fail to identify the right model, showing a tendency to include redundant variables in the selected solutions. To address this problem, a modified version of the BIC criterion is proposed, which, instead of assuming that the models have all the same probability, includes a penalty term for models with high redundancy in the predictor variables.

The proposed a priori probability distribution of the models depends on an overall quantity that we will indicate as WMRR (Weighted Mutual Regressor Relevance). Given a set of regressors $X_{1},X_{2},\ldots X_{N}$ and a dependent variable Y, $MRP_{XY}$ is defined as

(14) $$WMRR=n\cdot \overset{N}{\underset{i=1}{\sum }}\overset{N}{\underset{j=1}{\sum }}MI\left(X_{i},X_{j}\right)\cdot \left(1-RL_{N}\left(X_{i},Y\right)\right),\;\;\;\;\;for\;i\neq j;$$

where $MI\left(X_{i},X_{j}\right)$ is the mutual information estimate between the i-th and j-th predictor variables and $RL_{N}\left(X_{i},Y\right)=\frac{RL_{N}\left(X_{i},Y\right)}{\underset{j}{\max}\left(RL_{N}\left(X_{i},Y\right)\right)}$ is the relevance between the *i*-th predictor and the predicted variable normalized to the maximum value.

This quantity is higher for models which make use of predictors highly correlated between them and that at the same time have low relevance to the dependent variable.

Note that since $MI\left(X_{i},X_{j}\right)\geq 0\;\forall \;X_{i},X_{j}\;$, WMRR is also positively define.

The proposed a priori models’ probability density function is a Chi-squared distribution function that can then be written as

(15) $$p\left(M_{i}\right)=\frac{MI_{XY}^{\frac{k}{2}-1}\cdot e^{-\frac{WMRR}{2}}\;\;\;}{2^{\frac{k}{2}}\cdot \Gamma \left(\frac{k}{2}\right)},\;with\;k=2$$

where $\Gamma \left(\frac{k}{2}\right)=\left(1-\frac{k}{2}\right)!$. In this way, Models with WMRR=0 have the highest probability of being chosen, while models with greater WMRR are penalised.

Plugging (15) into (11) one obtains the following:

(16) $$p\left(Y|M_{i}\right)\approx L\left(Y|M_{i},\hat{\theta_{i}}\right)\cdot e^{-\frac{\left|\hat{\theta_{i}}\right|}{2}\log n}\cdot \;\frac{e^{-\frac{WMRR}{2}}\;\;\;}{2}$$

Which can be rewritten as

(17) $$2\cdot \log\left(p\left(Y|M_{i}\right)\right)\approx 2\cdot \log\left(L\left(Y|M_{i},\hat{\theta_{i}}\right)\right)-\left|\hat{\theta_{i}}\right|\cdot \log n-WMRR$$

Maximizing (17) is equivalent to minimizing

(18) $$MIBIC=-2\cdot \log\left(L\left(Y|M_{i},\hat{\theta_{i}}\right)\right)+\left|\hat{\theta_{i}}\right|\cdot \log n+WMRR\;\;\;$$

If, as it is often the case, the likelihood is difficult or impossible to calculate, and the variables are identically distributed and independently sampled from a normal distribution, the MIBIC can be written in the practical form:

(19) $$MIBIC=n\cdot \ln\left(\sigma_{\left(\epsilon \right)}{}^{2}\right)+k\cdot \log n+WMRR$$

where as usual *k* indicates the number of the model’s parameters.

The choice of the prior, which is a delicate point in any Bayesian statistical treatment, deserves a comment. Since WMRR is positive definite, its probability distribution function should also be supported on semi-infinite intervals [0, ∞). Moreover, since the main idea behind the proposed improvement of the criterion hinges on penalizing models with strongly correlated variables, this pdf should reach its maximum value when WMRR=0 and decrease as WMRR increases. There are several pdf that satisfy these conditions, but the Chi-squared distribution with *k* = 2 is the most uninformative in the exponential family. Indeed, its implementation implies the simplest WMRR linear correction term in the upgraded BIC.

### 3.2. Upgraded Version of the AIC

The derivation of the AIC criteria is based on the concept of minimizing the Kullbach–Leibler divergence between the model generating the data and the fitted candidate model. Given the different derivation approach compared to the BIC, the formal addition of an a priori probability distribution function of the models is not possible. Nevertheless, since the AIC is also based on the assumption that the independent variables have already been properly identified and that the effects of the measurement uncertainties are negligible, it is reasonable to include a correction term also in the AIC, which can help in the model selection process when these assumptions are not met. As a consequence, in analogy with the already described MIBIC, the following upgraded version of the AIC, called *MIAIC*, is proposed:

(20) $$MIAIC=n\cdot \ln\left(\sigma_{\left(\epsilon \right)}{}^{2}\right)+2\left|\hat{\theta_{i}}\right|+WMRR\;\;\;$$

It is worth mentioning that the same argument, leading to the same upgrade, is equally valid for the other indicators belonging to the AIC family, such as the c-AIC and the QAIC. Indeed, for the types of applications that are the subject of this work, these indicators can be expressed as the original AIC plus an additive term [6]. Consequently, perfectly analogue versions including the WMRR can be easily calculated and have proved to be at least equally effective.

## 4. Results of Systematic Tests with Synthetic Data

To evaluate the performance of the upgraded versions of the indicators developed in this work, a series of systematic tests have been performed. The main families of functions have been investigated: power laws, polynomials, exponentials, and combinations thereof.

Given the importance of the functional dependence and of the fact that the experimental case studied in the following belongs to this family, power laws are discussed first, which illustrates the methodology of the test in detail.

A synthetic dependent variable is generated from a set of predictor variables in the power-law form reported below:

(21) $$Y=\alpha_{0}\cdot X_{1}^{\alpha_{1}}\cdot X_{2}^{\alpha_{2}}\ldots \cdot X_{N}^{\alpha_{N}}$$

The predicted variable Y is generated with Equation (21) using 3 uncorrelated random predictor variables, $X_{1},\;X_{2},\;X_{3}$, from the $N\left(\mu =10,\sigma_{N}=1\right)$ distribution. The coefficients in (21) are all set equal to 1, and the number of data points generated is n=5000.

A fourth correlated predictor variable is added to the set of possible regressors of y in the form reported below:

(22) $$X_{4}=X_{1}+N\left(\left(\mu =0,\sigma_{N}=0.3\cdot std\left(X_{1}\right)\right)\right)$$

Then, a normally distributed noise $N\left(\mu =0,\sigma_{N}=\frac{noise\%}{100}\cdot std\left(X_{1}\right)\right);\;for\;i=1,\ldots ,4$ is added to all predictors. The parameter noise% is the percentage of noise with respect to the standard deviation of the regressor and is varied between 1% and 30%. A noise of the same type $N\left(\mu =0,\sigma_{N}=0.1\cdot std\left(Y\right)\right)$ is added to the independent variable Y.

After generating the variables and adding the noise, two models of the predicted variable fitting (20) to the noised values of Y have been obtained: The first using all the four noised predictors available, $X_{1},\;X_{2},\;X_{3},\;X_{4}$, and the second using only the noised predictors $X_{1},\;X_{2},\;X_{3},\;$ used to build y.

The two obtained models are compared using both the standard and the modified version of the AIC and BIC.

**Figure 1:**
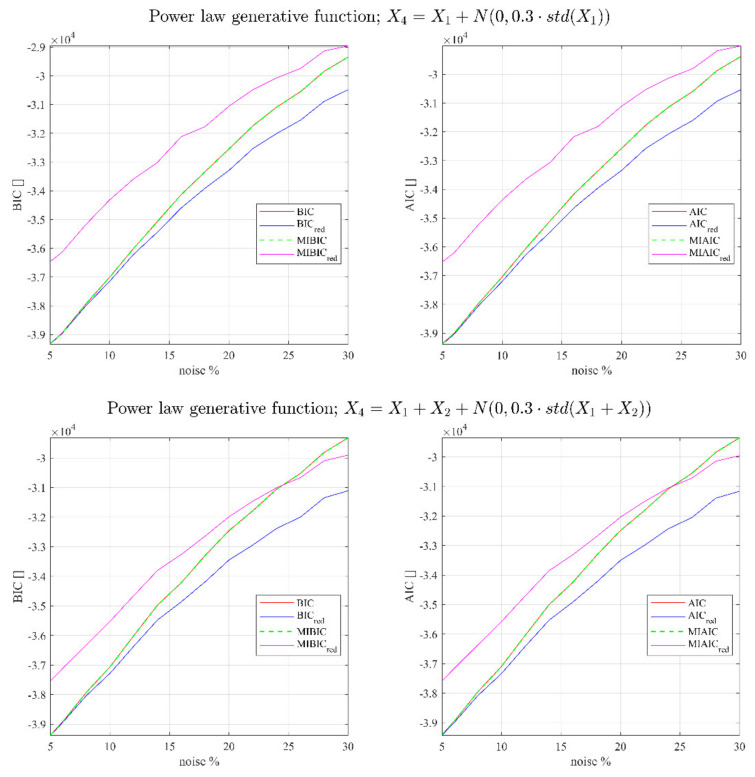

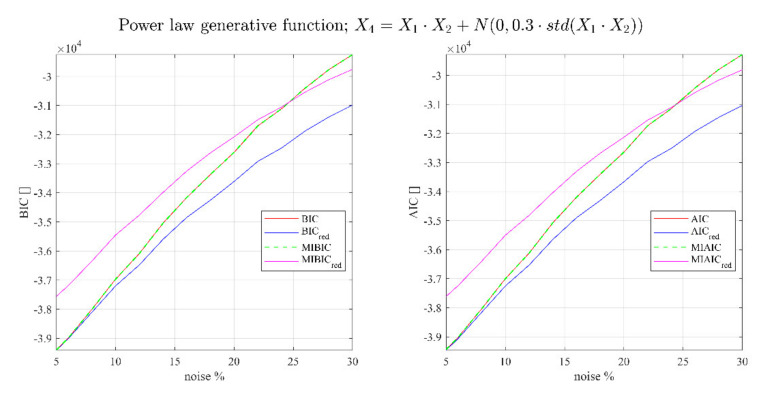
BIC, BICred, MIBIC, and MIBICred for the power-law generative function and different correlations as a function of the percentage of noise in the predictors. The subscript red indicates the models using the redundant regressors.

The results of the comparison varying the parameter noise% are reported in Figure 1. Each result reported in these plots is an average of over 5 repetitions of the calculations.

As can be noted from inspection of Figure 1, apart from the cases with very low noise, the model obtained, including the redundant variable, would always be chosen over the right model by the traditional AIC/BIC. Instead, the modified versions always succeed in identifying the right model.

The analysis has then been performed for other two types of correlation functions for the redundant variables:

(23) $$X_{4}=X_{1}+X_{2}+N\;\left(\mu =0,\sigma =\sigma_{N}=0.3\cdot std(X_{1}+X_{2})\right)$$

(24) $$X_{4}=X_{1}\cdot X_{2}+N\;\left(\mu =0,\sigma =\sigma_{N}=0.3\cdot std\left(X_{1}\cdot X_{2}\right)\right)$$

The results of the analysis are also reported in Figure 1.

The same analysis has been repeated for polynomial and exponential types of functions. The functions used to generate the data are the following, respectively:

(25) $$Y=X_{1}+X_{2}+X_{3}+X_{1}^{2}+X_{2}^{2}+X_{3}^{2}$$

(26) $$Y=X_{1}\cdot X_{2}\cdot X_{3}\cdot e^{X_{1}+X_{2}+X_{3}}$$

While the functions used to fit the data are respectively of the form

(27) $$Y=\alpha_{0}+\alpha_{1}X_{1}+\alpha_{2}X_{2}+\cdots +\alpha_{N}x_{N}+\alpha_{1}x_{1}^{2}+\alpha_{2}x_{2}^{2}+\cdots +\alpha_{N}x_{N}^{2}$$

(28) $$Y=\alpha_{0}\;\cdot X_{1}\cdot X_{2}\ldots \cdot X_{N}\cdot e^{\alpha_{1}X_{1}+\alpha_{2}X_{2}+\cdots +\alpha_{N}X_{N}}$$

**Figure 2:**
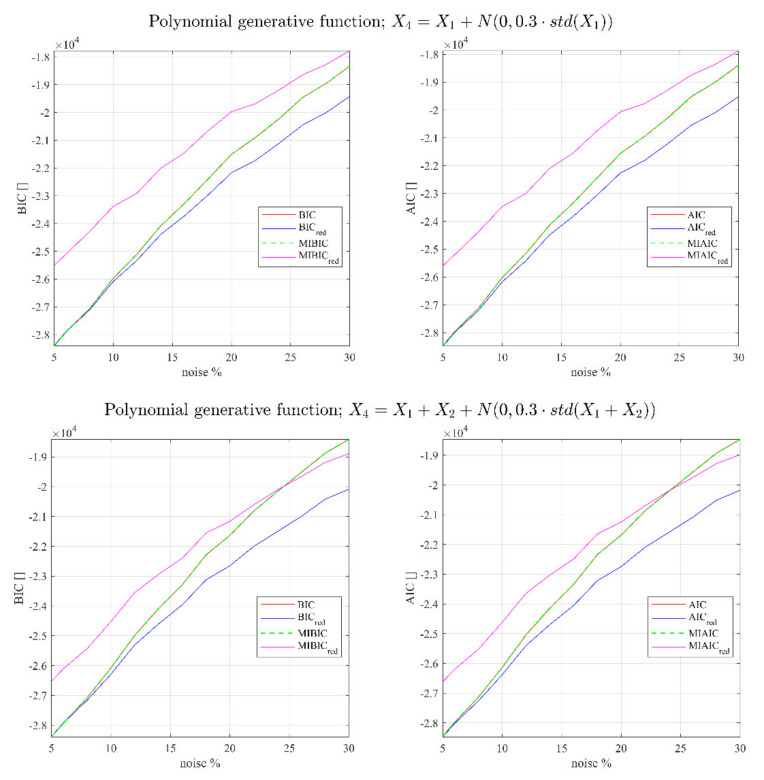

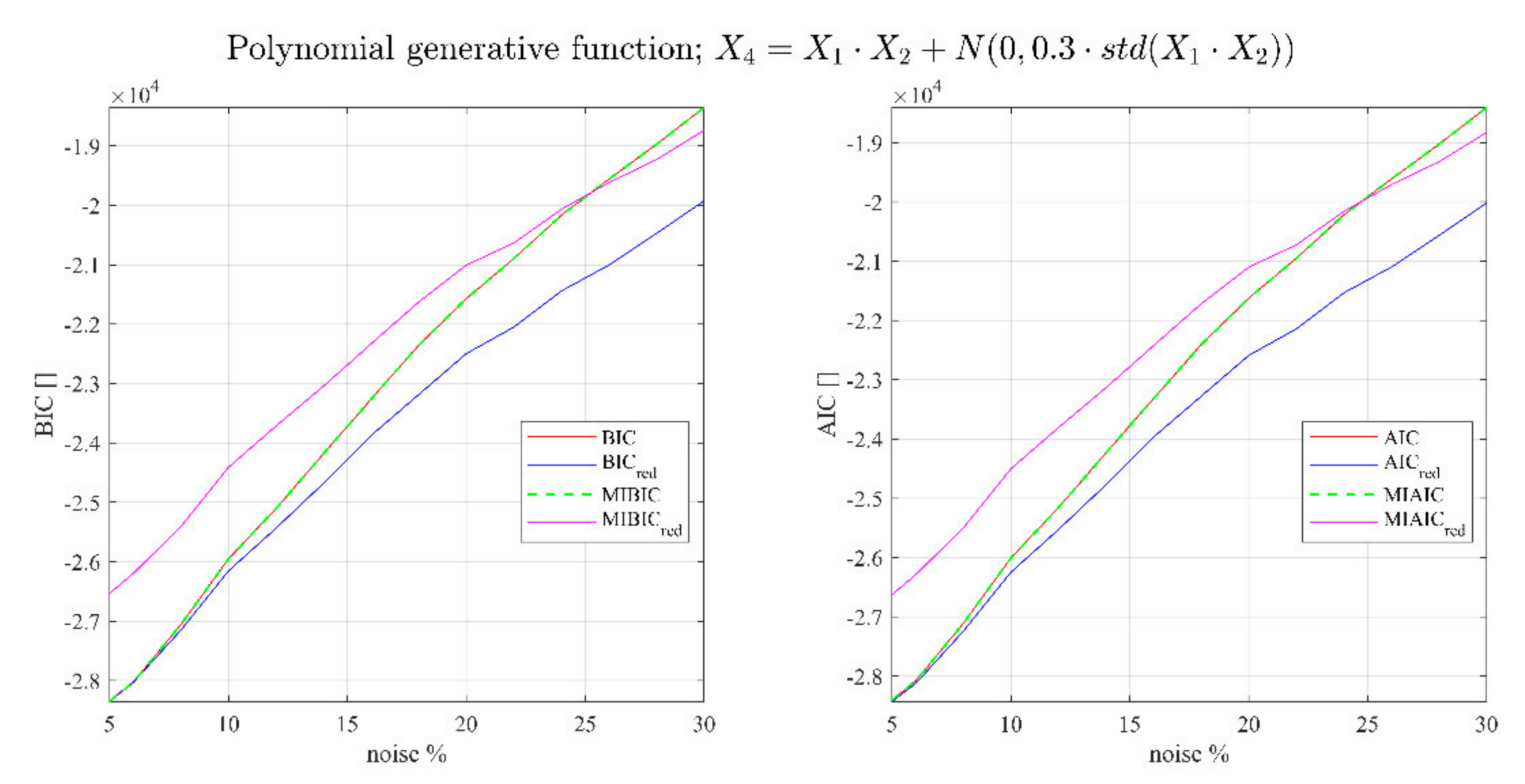
BIC, BICred, MIBIC, and MIBICred for the polynomial generative function and different correlations as a function of the percentage of noise in the predictors. The subscript red indicates the models using the redundant regressors.

**Figure 3:**
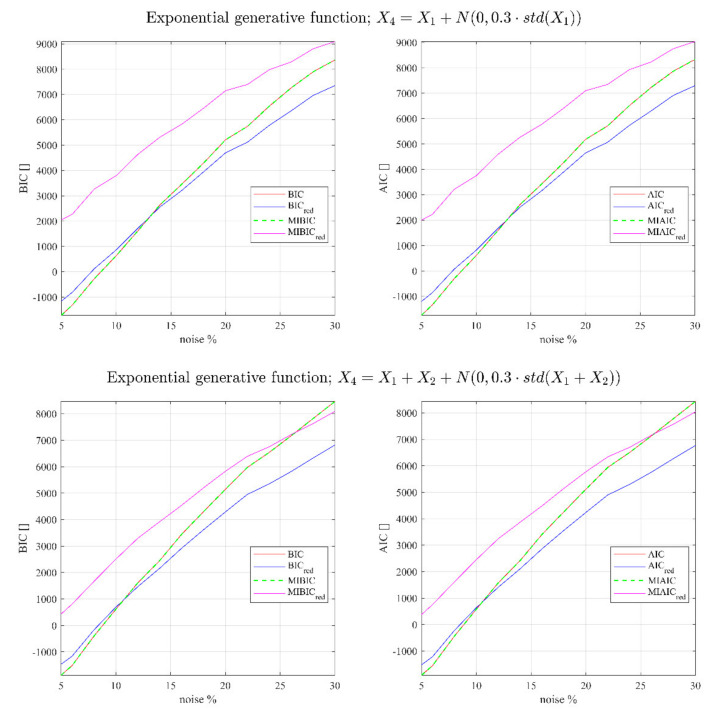

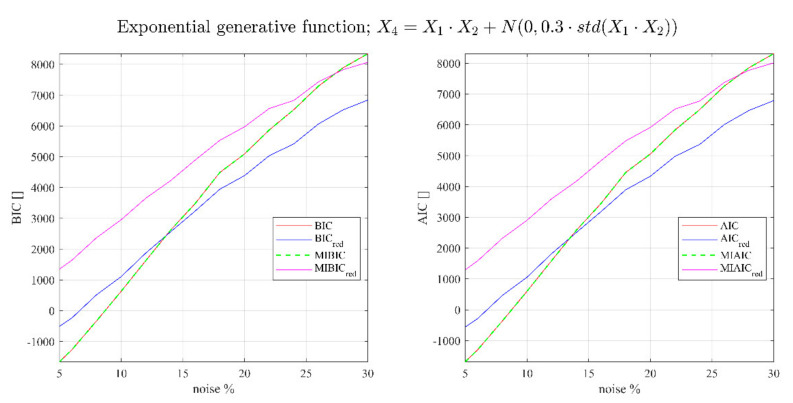
BIC, BICred, MIBIC, and MIBICred for the exponential generative function and different correlations as a function of the percentage of noise in the predictors. The subscript red indicates the models using the redundant regressors.

Fitting (27) and (28) to the noised values of Y with and without the redundant predictor and evaluating the traditional and the modified version of AIC and BIC, provides the results reported in Figure 2 and Figure 3. All the results shown have been obtained using n=5000 data points.

One important thing to notice in order to interpret the next figures is that without the redundant regressors, MIBIC and MIAIC provide exactly the same results as the traditional AIC and BIC, proving the consistency of the devised new indicators. On the other hand, if the redundant variables are added to the inputs, the traditional versions of the indicators would always select the wrong model (they assume a lower value), whereas the upgraded versions are not misled (the new indicators assume always higher values than when the redundant quantities are not considered).

In all the reported cases, except for small percentages of noise in the predictors, the traditional version of AIC and BIC are not able to identify the correct model, showing a tendency to select the models including redundant regressors. On the contrary, a significant improvement in the ability to detect the right model is achieved by the MIBIC as well as by MIAIC, which fails only in some cases when the noise percentage is significant.

The effect of the noise in the dependent variable has also been evaluated, but the results of the analysis are not significantly different and the conclusions are the same as for the examples reported.

## 5. Application to a Real-Life Database

In this section, to prove the generality of the results obtained with the upgraded criteria described in the previous sections, an application to a real-life database has been considered. The analysed database is called the ITPA database, which is the most advanced Multi-machine database built to support studies of plasma confinement in Tokamaks [10]. A description of this database is given in the next subsection. The results obtained applying the criteria developed in the present work to this database are reported in the following subsection.

### 5.1. The ITPA Database of the Energy Confinement Time for the H Mode

One of the most crucial quantities to assess the relevance of a nuclear fusion reactor is the so-called energy confinement time 𝜏𝐸, which quantifies how fast the internal energy of the plasma is lost [11,12,13]. Unfortunately, the transport mechanisms affecting the energy confinement in high-temperature plasmas are very complex and nonlinear, including effects at many scales. So, even if the understanding of the instabilities and turbulence effects influencing transport has progressed a lot in the last years, a theoretical or numerical solution for the proper estimation of the energy confinement time, $\tau_{E}$, remains unfeasible. As a consequence, this problem has been approached empirically with the extraction of robust scaling laws for 𝜏𝐸 from experimental data. This led to the construction of several multi-machine databases for the plasma confinement time, including the ITPA database analysed in this paper. In particular, the DB3v13f version of the ITPA with the same selection rules reported in [10] is the one used in the following analysis.

The variables that are known to be relevant for the estimation of the confinement time and that will be taken into consideration in this work are 𝐼𝑝, 𝐵𝑇, 𝑃𝐿𝑇𝐻, 𝑛𝑒𝑙, 𝑀𝑒𝑓𝑓, 𝑅𝐺𝐸𝑂, 𝜖, and 𝑘𝑎, where 𝐼𝑝 is the plasma current, 𝐵𝑇 is the toroidal magnetic field, 𝑃𝐿𝑇𝐻 is the power loss across the last closed surface, 𝑛𝑒𝑙 is the line average electron density, 𝑀𝑒𝑓𝑓 is the plasma isotopic composition, 𝑅𝐺𝐸𝑂 is the plasma major radius, $\epsilon =\frac{a}{R_{GEO}}$ where 𝑎 is the plasma minor radius, and 𝑘𝑎 is the volume measure of elongation [10]. Indeed, these variables are the ones used in the most widely accepted scaling law for the Tokamak energy confinement time in H mode, called the IPB98(y,2):

(29) $$\tau_{E}=5.62\cdot 10^{-2}\cdot I_{p}^{0.93}\cdot BT^{0.15}\cdot n_{e}^{0.41}\cdot P^{-0.69}\cdot R^{1.97}\cdot k_{a}^{0.78}\cdot \epsilon^{0.58}\cdot M_{eff}^{0.19}$$

Due to the physical constraints and the fact that each machine is optimized to work within specific parameter ranges, the degree of correlation of the mentioned regressors is quite high, as shown in Table 1.

Moreover, the regressors, as well as the confinement time, are affected by significant uncertainties, as shown in Table 2.

### 5.2. Results

Employing the upgraded model selection criteria proposed in this work, the main objective of the analysis consists of identifying, within all the possible power-law models obtained combining the predictor variables included in Equation (29), the one which best represents the $\tau_{E}$ data.

In order to do this, the following iterative procedure has been adopted:

The first step consists of evaluating the MIAIC and MIBIC for the power-law model with all the eight regressors included. Then, removing one variable at a time from the list of regressors, eight more models are obtained and their MIAIC and MIBIC evaluated.

The model with the lowest MIAIC/MIBIC is identified, and the regressors included in the model will form the new list of possible regressors. The process is then iterated eliminating one variable at the time, until removing any of the variables included in the list does not produce any benefit in terms of MIAIC and MIBIC. At this point, the algorithm is topped and the best model is retained

Applying this procedure, the model which shows itself to be the best in terms of both MIAIC and MIBIC is

(30) $$\tau_{E}=\alpha_{0}\cdot I_{p}^{\alpha_{1}}\cdot P^{\alpha_{2}}\cdot R^{\alpha_{3}}\cdot k_{a}^{\alpha_{4}}\cdot \epsilon^{\alpha_{5}}\cdot M_{eff}^{\alpha_{6}}$$

Instead, using the traditional AIC and BIC criteria, the resultant models are, respectively,

(31) $$\tau_{E}=\alpha_{0}\cdot I_{p}^{\alpha_{1}}\cdot BT^{\alpha_{2}}\cdot n_{e}^{\alpha_{3}}\cdot P^{\alpha_{4}}\cdot R^{\alpha_{5}}\cdot k_{a}^{\alpha_{6}}\cdot \epsilon^{\alpha_{7}}\cdot M_{eff}^{\alpha_{8}}$$

(32) $$\tau_{E}=\alpha_{0}\cdot I_{p}^{\alpha_{1}}\cdot BT^{\alpha_{2}}\cdot n_{e}^{\alpha_{3}}\cdot P^{\alpha_{4}}\cdot R^{\alpha_{5}}\cdot k_{a}^{\alpha_{6}}\cdot \epsilon^{\alpha_{7}}$$

The first obvious advantage of the upgraded versions of the criteria is that they provide coherent results, whereas the traditional versions of the indicators do not seem to agree on a single model, rendering the choice of the most appropriate scaling law very difficult. More importantly, the model obtained with MIAIC and MIBIC is more parsimonious, and indeed, it utilises fewer quantities than the ones derived by the AIC and IC. It retains the plasma current but considers redundant magnetic field and plasma density. This is coherent with the statistical analysis of the database, which presents very strong collinearities between these three quantities, as reported in Table 1. The obtained results are also in harmony with the everyday experience of the device operators, since the experiments are indeed typically designed with strong correlations between plasma parameters.

## 6. Conclusions

In applications to regression, the most widely used versions of the model selection criteria AIC and BIC are vulnerable to the presence of variables correlated to the actual predictors, particularly when the percentage of noise in the regressors is not negligible, as it is in most practical applications. To address this problem, an upgraded version of these criteria is proposed, adding an a priori Chi-squared probability distribution function of the models. This function depends on a quantity that penalizes the model with highly correlated predictors, which bring little new information about the dependent variable. The performance of the proposed criteria has been assessed with different types of generative functions, correlation functions and percentage of noise in the predictors. The results indicate that, in most cases, the newly defined criteria possess an improved capability of detecting redundancy in the predictors and thus of selecting the correct model. The improved performances are not substantially affected by the sample size as reported in Appendix A. To show the generality of the obtained results, an application to an international database built by the thermonuclear fusion community has also been reported in the final section.

With regard to future developments, from a methodological standpoint, it would be interesting to improve the treatment of the uncertainties in both the dependent and independent variables, implementing techniques inspired by the error in the variable approach [10]. Moreover, the introduction of metric alternatives to the Euclidean, such as the geodesic distance [14,15,16], has the potential to provide significant added value. An additional interesting activity would be the systematic analysis of possible prior alternatives to the one chosen for the present version of MIBIC and MIAIC. In terms of applications, the scaling laws of the more recent metallic Tokamaks, and particularly JET with the new ITER-like wall [17], are nowadays a topic of great interest in the fusion community. The new versions of the indicators could become quite useful in the investigation of scaling laws in non-power law monomial form [18,19,20,21].

## Figures and Tables

**Table 1:** Person correlation coefficient matrix for the eight regressors used to model the energy confinement time.

	ϵ	$M_{eff}$	$R_{GEO}$	$k_{a}$	$B_{T}$	$I_{P}$	$n_{e}$	$P_{LTH}$
ϵ	1.00	0.29	0.11	0.41	−0.02	0.41	0.10	0.26
$M_{eff}$	0.29	1.00	0.30	0.42	0.17	0.38	0.16	0.36
$R_{GEO}$	0.11	0.30	1.00	0.30	0.09	0.73	−0.36	0.67
$k_{a}$	0.41	0.42	0.30	1.00	−0.07	0.48	0.15	0.42
$B_{T}$	−0.02	0.17	0.09	−0.07	1.00	0.43	0.52	0.34
$I_{P}$	0.41	0.38	0.73	0.48	0.43	1.00	0.00	0.76
$n_{e}$	0.10	0.16	−0.36	0.15	0.52	0.00	1.00	0.03
$P_{LTH}$	0.26	0.36	0.67	0.42	0.34	0.76	0.03	1.00

**Table 2:** Lower bounds of the uncertainties for the entries of the ITPA database.

	ϵ	$M_{eff}$	$R_{GEO}$	$k_{a}$	$B_{T}$	$I_{P}$	$n_{e}$	$P_{LTH}$	$\tau_{E}$
Rel. err.	1%	8%	1%	10%	1%	1%	5%	14%	10%

**Figure A1:**
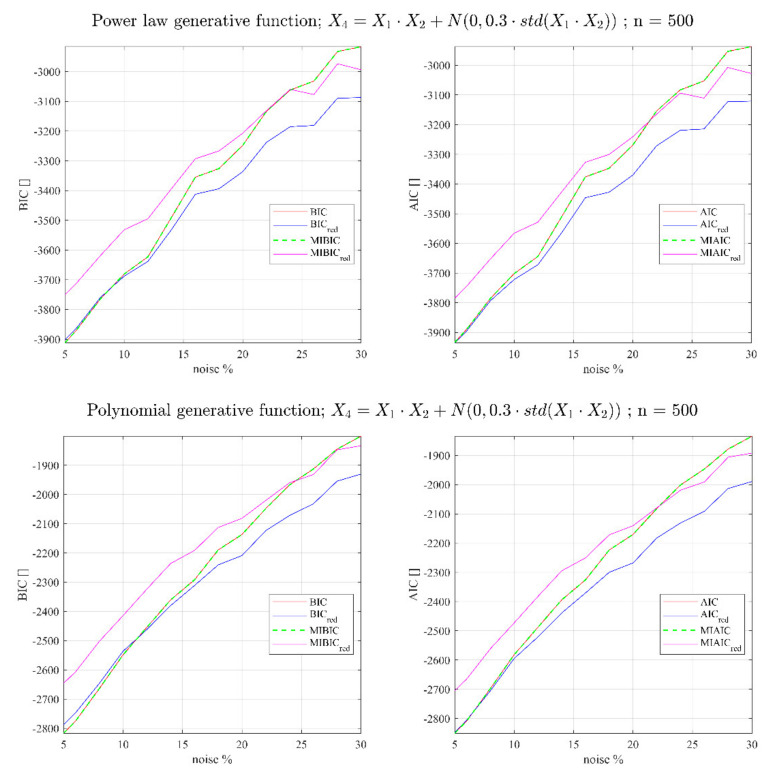

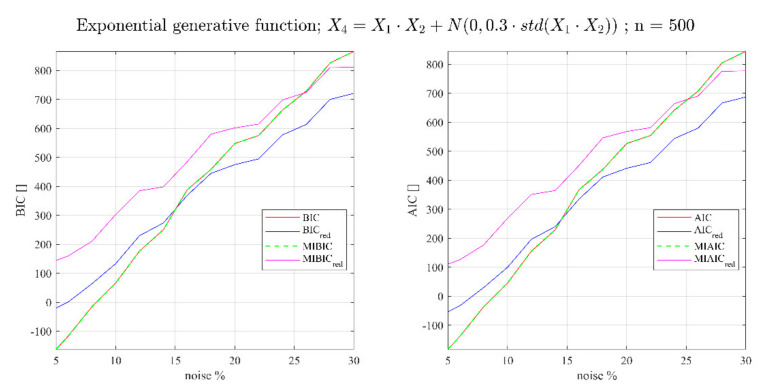
BIC, BICred, MIBIC, MIBICred for *n* = 500 and different generative functions vs. the percentage of noise in the predictors. The subscript red indicates the models using the redundant regressors.

**Figure A2:**
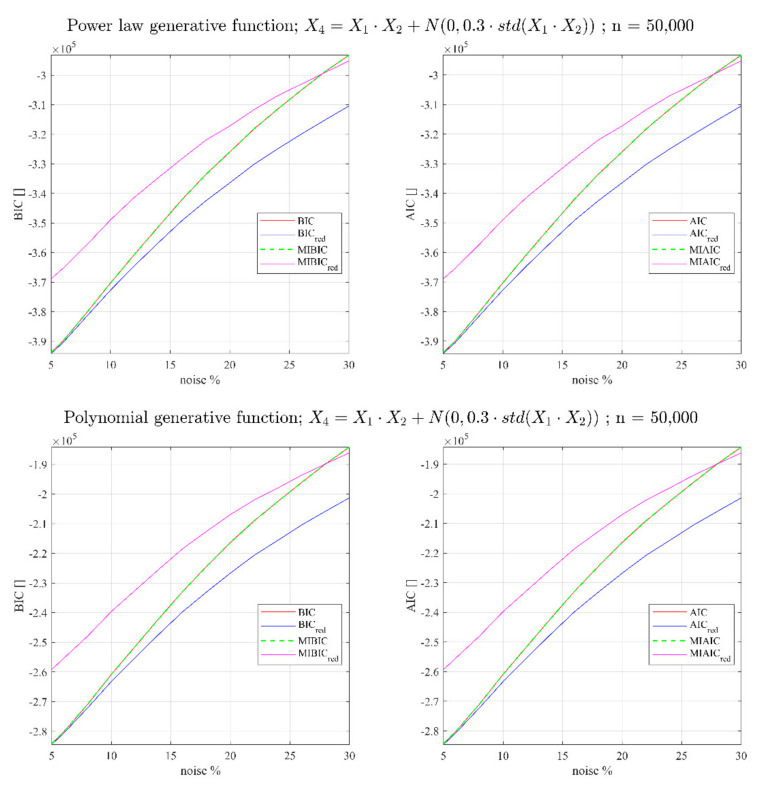

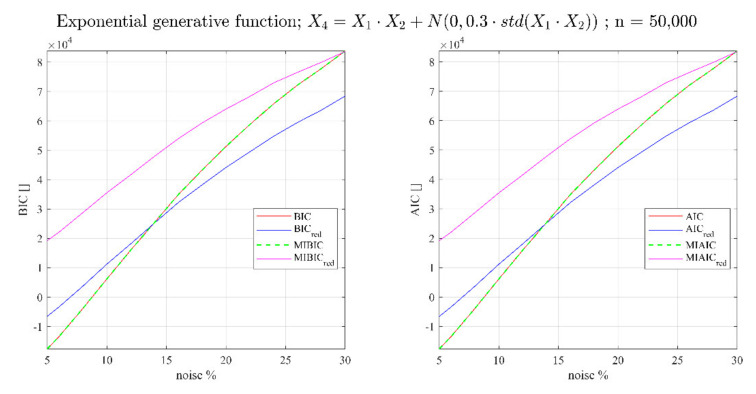
BIC, BICred, MIBIC, MIBICred for *n* = 50000 and different generative functions vs. the percentage of noise in the predictors. The subscript red indicates the models using the redundant regressors.

## Data Availability

The data presented in this study are available on request from the corresponding author.

## Appendix A. MIBIC and MIAC Performance with Sample Size

The Appendix A contains the analysis of the effect of the number of samples on the performance of the proposed upgraded criteria. In particular, the same tests reported in Section 4 have been repeated for two different sample sizes n=500 and n=50,000. The results of the analysis for the different generative functions and number of entries are reported in Figure A1 and Figure A2. For simplicity, only the results for the correlation function reported in (24) have been included in the figures. As it can be noted by visual insèecion of the plots reported in this Appendix A, the performance’s improvement associated with the proposed criteria is not affected by the sample size. This is a general result: the MIBIC and MIAIC perform better than the traditional versions independently from the number of entries in the datbases. These results can be also extended to the other correlation functions considered in Section 4, even though the related figures have not been explicitly reported in the paper.
